# Training human super-recognizers’ detection and discrimination of AI-generated faces

**DOI:** 10.1098/rsos.250921

**Published:** 2025-11-12

**Authors:** Katie L. H. Gray, Josh P. Davis, Carl Bunce, Eilidh Noyes, Kay L. Ritchie

**Affiliations:** ^1^ School of Psychology and Clinical Language Sciences, University of Reading, Reading, UK; ^2^ School of Human Sciences, Institute of Lifecourse Development, University of Greenwich, London, UK; ^3^ School of Psychology, University of Leeds, Leeds, UK; ^4^ School of Psychology, Sport Science & Wellbeing, University of Lincoln, Lincoln, UK

**Keywords:** AI-generated faces, super-recognizers, synthetic faces, training

## Abstract

Generative adversarial networks (GANs) can create realistic synthetic faces, which have the potential to be used for nefarious purposes. The synthetic faces produced by GANs are difficult to detect and are often judged to be more realistic than real faces. Training programmes have been developed to improve human synthetic face detection accuracy, with mixed results. Here, we investigate synthetic face detection and discrimination in super-recognizers (SRs; who have exceptional face recognition skills), and typical-ability control participants. We also devised a training procedure which sought to highlight rendering artefacts. In two different experimental designs, we found that SRs (total *N* = 283) were better at detecting and discriminating synthetic faces than controls (total *N* = 381), where control participants were below chance without training. Trained SRs and controls had significantly better performance than those without training, and the magnitude of the training effect was similar in both groups. Our results suggest that SRs are using cues unrelated to rendering artefacts to detect and discriminate synthetic faces, and that an easily implementable training procedure increases their performance to above chance levels. These results have implications for real-world scenarios, where trained SRs' performance could be harnessed for synthetic face detection.

## 1. Introduction

Synthetic faces—faces that are completely artificial intelligence (AI)-generated—are now extremely realistic (e.g. [1]), to the extent that human observers are highly error-prone at discriminating them from real faces (e.g. [2–4]). This poses a problem, as synthetic faces have the potential to be used for nefarious purposes in various domains, including digital security, social media and forensic investigations. For example, synthetic faces have been used to give credence to fake social media accounts which have then been used to harass targets on social media [5].

Synthetic faces are generated through generative adversarial networks (GANs; [6–8]). To achieve this, GANs pit two networks against each other, one that generates face-like images and the other that discriminates them from real faces. Over time, the generating network learns to create faces that look more and more realistic. The synthetic faces produced by GANs seem to share some underlying processing mechanisms with real faces [9]. They are not only difficult for humans to detect, but there is research to suggest that people judge these synthetic faces to be more trustworthy [3], and more realistic [1,3,4,10] than real faces. The latter has been coined *AI hyperrealism* [1]. Miller *et al*. [1] have suggested that AI hyperrealism exists because synthetic faces are perceived as more ‘average’, more familiar, and less memorable than real faces, which misleads people to perceive synthetic faces as real. ‘Averageness’ is thought to be a consequence of the GAN production process [1]. One model of face perception suggests that real faces are represented in a multidimensional space (i.e. face-space [11,12]), where the average face is situated in the middle of the space, and distinctiveness is represented as distance from the average on any particular dimension [11–13]. For real faces, because each dimension is thought to follow a normal distribution, there is a statistical over-representation of features near the middle (or ‘average’) of face space [11,12]. This over-representation of features near the average is reinforced during the generative learning process.

Training programmes have been used to increase human detection accuracy of synthetic images, with varying success [3,14–16]. For example, Nightingale & Farid [3] gave typical-ability participants a short training procedure to highlight possible synthetic face rendering artefacts, as well as trial-by-trial feedback throughout the experiment. They found an increase of approximately 10% in participants’ ability to classify images as real or not-real following training. Bray *et al*. [14] developed three training conditions in an attempt to increase individuals’ ability to detect synthetic faces and compared participants’ detection accuracy in the three training methods against a control group with no training. The training methods were: familiarization, where participants were shown synthetic images that were labelled as such; one-time advice, where participants were given information on the types of rendering problems that might occur with synthetic faces at the start of the experiment; and advice with reminders, where participants were given advice on the types of rendering problems to expect, with reminders throughout the experiment. They found that each group of participants was at around 60% accuracy, with no statistically significant differences between the training programmes and the control group (51% accuracy).

Another suggested method of improving synthetic face detection, borrowed from the literature on the identification of real faces, is to use a ‘wisdom of the crowds’ approach whereby responses from multiple observers are combined [17–19]. The success of this approach for the detection of synthetic faces is mixed, with Kramer & Cartledge [2] reporting improvements, but with Dunn *et al.* [20] finding no advantage for synthetic face detection in typical observers. There is also a large effort from the computer science literature to address this problem by training AI to detect synthetic faces (e.g. [21]). In applied practice, a human-in-the-loop approach (where human(s) are integrated into the algorithm decision-making pipeline) is often used for the identification of real faces. As synthetic faces become more commonplace, we expect that the detection of synthetic faces will also include both humans and face recognition algorithms. Typical human observers are now worse at face matching (determining whether two images show the same person or not) than top-performing algorithms, and the human in the loop can introduce errors (e.g. [22]). One solution that has been applied to forensic face identifications for real faces (e.g. [23]) has been to exploit the capabilities of individuals who have exceptional face recognition ability, known as ‘super-recognizers’ (SRs). It is yet to be seen if SRs’ skills extend to the detection of synthetic faces.

SRs are people who perform well above typical levels on a range of face perception and recognition tasks ([24]; see [25,26] for reviews). Their responses are described as fast and intuitive [27], and as a group, they have outperformed typical-ability control participants on perceptual face identity processing tasks [28–31], face memory tasks [28,29,31,32], tasks involving occluded faces [33,34], detection of hyper-realistic face masks [35] and morphed/other digitally manipulated images [36]. Currently, few studies have investigated SRs’ ability to detect synthetic faces. One such study focussed on dynamic ‘deep fake’ videos and showed no difference between SRs’ and controls’ accuracy [37]. A second study investigated synthetic face detection [20]. This recent synthetic face detection experiment suggested that SRs were better able to detect faces generated by StyleGAN2 than control participants [20]. This study showed that SRs’ detection of StyleGAN2 faces was significantly above chance, and that their confidence correlated with their accuracy.

Here, we investigated whether SRs were better able to detect synthetic faces than typical-ability control participants and investigated whether a training procedure could enhance performance. Our synthetic faces were generated using StyleGAN3 [38]. StyleGAN3 was the state-of-the-art system at the time of data collection and supersedes StyleGAN2, which has been used in previous research on synthetic face detection [1–3,20]. In experiment 1a, we asked participants to make a binary response, indicating whether a singular, centrally presented face was AI-generated (‘not real’ or ‘real’). In experiment 1b, we trained a new sample of participants to detect synthetic faces and gave them feedback before testing their synthetic face detection ability in the same forced-choice paradigm. In this forced-choice procedure, we used signal detection theory to model the differences in sensitivity and bias related to participant group and training. In experiment 2a, we conducted a two-alternative forced choice (2AFC) task, where participants were required to indicate which of two simultaneously presented faces was the AI-generated (‘not real’) face. In experiment 2b, a new sample of participants was trained to discriminate synthetic faces and given feedback before completing the 2AFC task. We used this 2AFC task to investigate whether participants’ performance was better when a direct comparison image was presented simultaneously. The 2AFC task has an additional benefit: because both stimuli are presented simultaneously and participants are required to choose one image, the effects cannot be driven by decisional biases.

We predicted that (i) SRs would be better at detecting and discriminating synthetic faces than typical-ability controls, (ii) our training procedure would improve synthetic face detection and discrimination in both groups, and (iii) the effects would be driven by differences in sensitivity, rather than decisional bias. We predicted that if SRs were better than control participants because they identify rendering artefacts, they may benefit less from training than control participants. On the other hand, if SRs use other cues to identify synthetic faces, they may benefit as much or more from training than control participants.

## 2. Experiment 1

### 2.1. Methods

#### 2.1.1. Participants

We recruited three groups of participants for the non-training experiments (SRs, prolific controls and database controls), and two groups of participants for the training experiments (SRs and prolific controls). SRs and database controls were invited from the Greenwich Face and Voice Recognition Laboratory volunteer database and had previously completed three to four screening tests at the time of invite, including the Cambridge Face Memory Test Long form (CFMT+; [24]), and the Glasgow Face Matching Task (GFMT; [39]) and at least one of the Kent Face Matching Task (KFMT; [40]) and the Adult Face Memory Test (AFMT; [41]; table 1). The majority of participants self-identified as White (SRs = 90.5%, prolific controls = 86.9%, database controls = 77.4%), followed by mixed (SRs = 4.8%, prolific controls = 3.3%, database controls = 9.7%), Black (SRs = 0%, prolific controls = 9.8%, database controls = 1.6%) and Asian (SRs = 0%, prolific controls = 0%, database controls = 8.1%). A small number of participants (SRs = 4.8%, prolific controls = 0%, database controls = 3.2%) selected ‘other’ or preferred not to disclose their ethnicity. All participants gave informed consent, and ethical clearance was granted by the local ethics committee (project code: 2024-016-KG).

**Table 1.** Group demographics and performance on face perception and recognition ability screening tests for the final samples. The number of participants (*n*) on a particular test is given if they differ from the overall *N*. CFMT+, Cambridge Face Memory Task Long form [24]; GFMT, Glasgow Face Memory Task [39]; KFMT, Kent Face Matching Task [40]; AFMT, Adult Face Memory Test [41]. ***p < 0.001; ** *p* < 0.01; * *p* < 0.05; n.s.: non-significant. Across tasks, the SR groups were well-matched. SRs in the no-training task were significantly better at the GFMT and AFMT than those in the training task (*p*s < 0.02); all other effects were non-significant. The prolific control groups were also well-matched across tasks (all effects non-significant).)

		*N*	gender	age (s.d.)	CFMT+ (s.d.)	GFMT (s.d.)	KFMT (s.d.)	AFMT (s.d.)
forced- choice task	super-recognizers (SR)	63	F: 36 M: 26 other: 1	42.11 (8.00)	96.83 (1.80)	39.83 (.46)	34.30 (1.94)	55.32 (2.31)
	prolific controls (PC)	61	F: 36 M: 25	38.30 (12.12)	—	—	—	—
	database controls (DC)	62	F: 44 M: 17 other: 1	44.84 (10.55)	75.02 (8.64)	34.02 (2.21)	26.69 (2.27)	44.52 (3.15)
	between group difference		n.s.	DC > PC **	SR > DC ***	SR > DC ***	SR > DC ***	SR > DC ***
forced-choice training	super-recognizers (SR)	76	F: 53 M: 23	40.57 (8.74)	96.00 (3.12)	39.43 (.85)	*n* = 75 34.16 (2.52)	*n* = 74 53.96 (4.06)
	prolific controls (PC)	70	F: 35 M: 32 other: 3	38.49 (9.90)	—	—	25.51 (3.82)	—
	between group difference		SR > PC *	n.s.	—	—	SR > PC ***	—

##### 2.1.1.1. Super-recognizers group

To be eligible for invitation SRs were required to have previously scored 2 s.d. above the mean on at least three of the above-listed tests, and at least 1.5 s.d. above the mean on the fourth test if they had completed a fourth test. SRs were not compensated for their time.

##### 2.1.1.2. Database control group

These were individuals who had participated in previous face processing experiments and had indicated that they would like to be contacted for future experiments. Database controls were invited if they scored between +1.0 and −1.5 s.d. from the mean on three tests, and between +1.5 and −2.0 s.d. on a fourth test, if they had completed a fourth. Note a slight asymmetry in the scores around the mean; this was put in place to balance the tendency of the database control participants to score above the mean generally (e.g. [20]). Database controls were not compensated for their time. Both the SR group and the database control group were able to complete additional screening tests between invite and data collection finalization. Any additional test scores collected subsequently to the invite are presented in our data repository (https://osf.io/4pv8f), alongside the test scores for each individual across the range of screening tests collected.

##### 2.1.1.3. Prolific control group

Finally, we recruited a control group from Prolific.co. For the non-training tasks, we did not collect face processing screening data for this group. For the training tasks, prolific controls also completed the KFMT [40]. They were compensated £3 or £3.50 for their time on the non-training and training tasks, respectively, and were required to be UK residents with English as a first language, and aged 18–60.

Previous research has indicated that the difference between SRs and controls’ synthetic face detection ability has a medium effect size (Cohen’s *d* = 0.55 [20]). We calculated that a sample size of 60 in each condition was required when running a one-way ANOVA with three independent groups, with a medium effect size (*f* = 0.25), and power of 0.85 (G*Power 3 [42]). Therefore, we collected at least 60 participants per condition.

### 2.1.2. Stimuli

**Figure 1.**
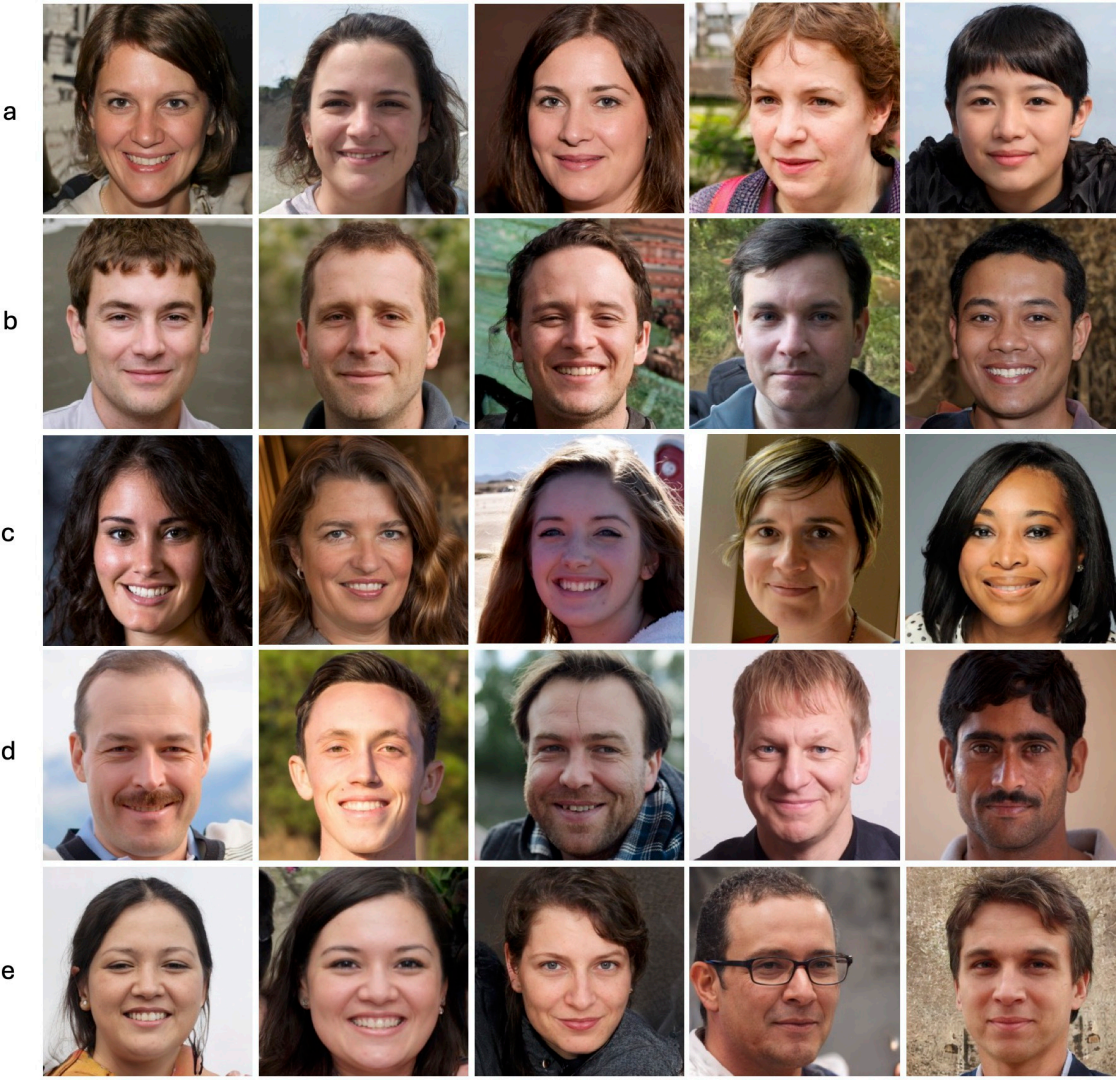
Example stimuli. Female synthetic faces (a), male synthetic faces (b), female real faces (c) and male real faces (d). The final row (e) contains some of the synthetic images used in the training task, each of which has rendering artefacts that were highlighted to participants. For example, the first image has hair that is poorly rendered, and the second image has three, rather than four incisors.

Our real faces were taken from the Flickr-Faces-HQ Dataset (FFHQ [7]; https://github.com/NVlabs/ffhq-dataset; figure 1). This is a set of high-quality face images taken from the website Flickr.com under appropriate licenses and originally used as a benchmark image set for StyleGAN synthetic faces [7]. We selected 80 faces from the set (40 judged by the authors to be male and 40 judged to be female). Thirty of the male and female faces were selected to be White, with the final 10 selected to be non-White, again as judged by the authors. All images included only one person (i.e. no other people pictured in the background of the image), all were selected to have no occlusions such as hats, glasses or hands over the face.

Our synthetic faces were generated using StyleGAN3 ([38]; https://github.com/NVlabs/stylegan3; figure 1). StyleGAN3 was trained using the FFHQ dataset [7] a demographically diverse set of face images that we used for our real faces, and the MetFaces dataset ([8]; https://github.com/NVlabs/metfaces-dataset), a set of artworks from the Metropolitan Museum of Art. Full details of the training process for StyleGAN3 can be found in the original paper and the accompanying GitHub site ([38]; https://github.com/NVlabs/stylegan3). We selected 80 StyleGAN3 faces which fitted the same demographic constraints as outlined above for the real faces (i.e. 40 judged to be male, 30 of which were judged to be White), and the same image constraints as the real faces (one person, no occlusions). Both our real and synthetic images were broadly front-facing and were cropped to be 1024 × 1024 pixels.

In addition to the experimental stimuli, four real and four synthetic faces were used for practice trials—none of these images were used in the experimental trials. An additional six synthetic faces were selected for the training task. These images all had rendering artefacts to the face. We selected another 10 real and 10 synthetic faces for the feedback component of the training task.

We investigated the low-level properties of the stimuli, comparing the synthetic and real images’ colour saturation, brightness and contrast. We computed these parameters individually for each synthetic (*n* = 80) and real (*n* = 80) face image using the SHINE_color toolbox [43]. Saturation, reflecting the vividness or purity of colour, was computed as the mean saturation value across pixels, ranging from 0 (fully desaturated/grey) to 1 (fully saturated). Brightness, reflecting the overall lightness of the image, was defined as the mean of the maximum red, green, blue (RGB) channel intensity at each pixel, ranging from 0 (black) to 255 (maximum brightness). We obtained a measure of contrast by calculating the s.d. of these pixel intensities, with higher values reflecting greater variability in brightness across the image.

Independent samples *t-*tests were conducted to assess whether saturation, brightness and contrast differed between the synthetic and real face image sets. A significant difference was observed for saturation. The real face images (*M* = 0.36, s.d. = 0.12) were found to be more saturated than the synthetic face images (*M* = 0.33, s.d. = 0.08), (*t*_158_ = 2.312, *p* = 0.022, *d* = 0.619). We observed no significant difference in brightness between the image sets (real: *M* = 138.89, s.d. = 29.82; synthetic: *M* = 134.85, s.d. = 20.06; (*t*_158_ = 1.005, *p* = 0.316, *d* = 0.313)). There was also no significant difference in contrast between the image sets (real: *M* = 63.95, s.d. = 12.04; synthetic: *M* = 61.37, s.d. = 10.73; (*t*_158_ = 1.431, *p* = 0.154, *d* = 0.226)).

### 2.1.3. Design

Experiment 1 consisted of a forced-choice experiment, where one stimulus was presented per trial, and participants indicated whether it was ‘real’ or ‘not real’. In the training version of the task, the identical experiment was run, but first, participants were given a short training and feedback session. All experiments described were conducted online. Carefully designed online tests of cognitive and perceptual processing can yield high-quality data, indistinguishable from that collected in the laboratory [44,45]. The experiments were conducted using Gorilla Experiment Builder, a cloud-based research platform that allows researchers to create and deploy experiments online and collect precise behavioural data [46]. Participants were instructed to use only desktop computers or laptops and to complete the experiment in a single session.

### 2.1.4. Procedure

Forced-choice task: to begin each trial, a fixation cross was presented centrally for 500 ms. Faces were presented individually; participants were asked to respond whether the face was ‘real’ or ‘not real’. Trials timed out after 10 s, and stimuli were presented for the whole duration unless a response was given. The experiment was separated into six blocks, with the first block constituting the practise trials; the first block was preceded by a set of instructions, including how to respond to catch trials. Four practise trials were given at the start of the experiment (two real, two synthetic faces), and one practise catch trial. Five catch trials were presented throughout the blocks (one in each additional block), where participants were asked to withhold their response (i.e. not respond) if they saw an object that was not a face, e.g. a chair or a lamp.

Forced-choice training: prior to completing the main task, participants were given a short training session. First, they were given examples of the type of artefacts that can be used to identify computer-generated faces (as in [3] with synthetic faces and [15] with deepfakes). The synthetic images in the training trials all had artefacts in the images which were drawn to the attention of the participants via the following text ‘When computers generate faces, sometimes there are tell-tale signs left in the images. Here are some examples of computer-generated faces with different things “wrong” with them’. The images were presented individually with additional text pointing out the specific artefacts in each, e.g. ‘This face has a “middle tooth”’, and ‘Where this person’s hair meets their face, it looks odd’. None of these images was re-used in any part of the experiment. Participants then completed 10 trials in a forced-choice design and were given feedback on their accuracy after each trial. Following the feedback trials, participants were again shown the artefact examples. The training and feedback procedure took around 5 min to complete.

### 2.1.5. Data analysis

Participants were included if they completed at least 3 out of 5 catch trials accurately. In the non-training task, 27 SRs, 13 database controls and 13 prolific controls were excluded on this basis. In the training task, seven SRs and 12 prolific controls were excluded on this basis. The participants described in table 1 are the final samples, after these exclusions had taken place. We report accuracy using percentage correct, and we also assessed sensitivity using the signal detection theory framework. Sensitivity (*d'*) and criterion (*C*) were calculated using the Palamedes toolbox [47] in MATLAB, where a hit was defined as correctly identifying a synthetic face as ‘not real’ and a false alarm was defined as incorrectly responding that a real face was ‘not real’. Median reaction times were calculated per participant and then averaged at the group level and are reported rounded to the nearest millisecond. Effect sizes are given as Cohen’s *d* (*t*-tests) or partial eta squared (ANOVA). Where equal variances were not assumed, degrees of freedom were corrected. Post-hoc *t*-tests were Bonferroni-corrected.

### 2.2. Results

#### 2.2.1. Forced-choice task

Synthetic face detection accuracy (i.e. hits) on the task was low for all three groups: SRs = 41% (s.d. = 23%), prolific controls = 31% (s.d. = 18%) and database controls = 30% (s.d. = 20%).

**Figure 2.**
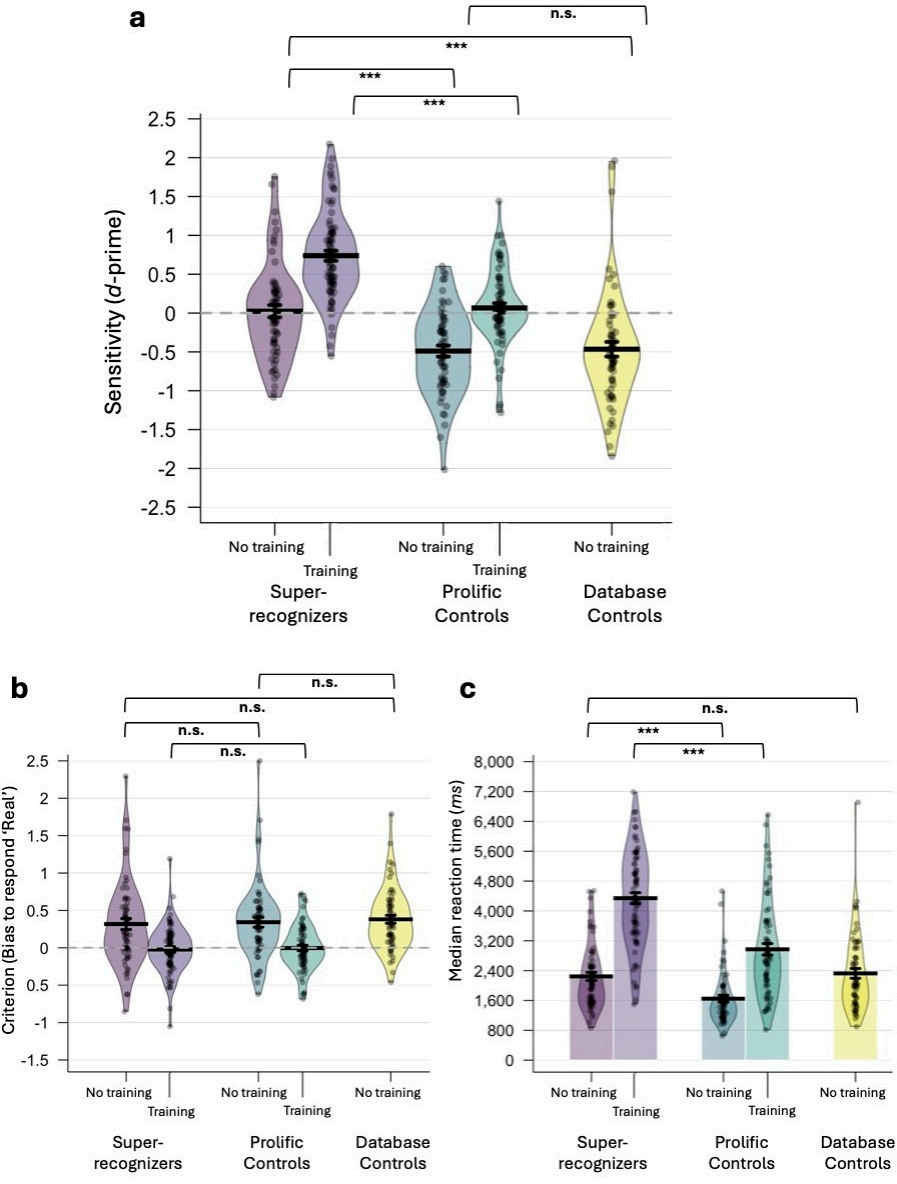
Sensitivity (a), decisional biases (b) and response times (c) for the forced-choice task and the forced-choice training task across the five participant groups. Note that the no-training and training tasks were run in independent sets of participants. ***p < 0.001; n.s.: non-significant. Error bars: standard error of the mean; dashed line: chance performance.

Given that participants’ decisional strategies could affect their accuracy, we analysed their sensitivity (*d'*; figure 2a), which is calculated from hits (as above) and false alarms (real faces categorized as synthetic; descriptive statistics for hits and false alarms are given in the electronic supplementary material, S1). There was a significant difference in sensitivity scores between the groups (*F*_2,183_ = 12.509, *p* < 0.001, *ηp*^2^ = 0.120). SRs (*M* = 0.024, s.d. = 0.627) were significantly more sensitive than both prolific controls (*M* = −0.489, s.d. = 0.557; (*t*_122_ = 4.809, *p* < 0.001, *d* = 0.864)) and database controls (*M* = −0.466, s.d. = 0.742; (*t*_123_ = 3.982, *p* < 0.001, *d* = 0.712)). No significant difference was found between the control groups (*t*_121_ = 0.201, *p* = 1.00, *d* = 0.036).

SRs’ sensitivity did not differ significantly from chance (i.e. *d'* = 0) (*t*_62_ = 0.299, *p* = 0.766, *d* = 0.038), indicating that they were not reliably distinguishing synthetic from real faces—but crucially, they also did not show evidence of AI hyperrealism once response biases were controlled for. By contrast, both the prolific (*t*_60_ = 6.861, *p* < 0.001, *d* = 0.878) and database (*t*_61_ = 4.939, *p* < 0.001, *d* = 0.627) controls had sensitivity scores significantly worse than chance performance, suggesting a role for AI hyperrealism in their responses.

To further investigate decisional strategies, we next analysed criterion (*C;* figure 2b). There was no significant difference between the groups (*F*_2,183_ = 0.243, *p* = 0.785, *ηp*^2^ = 0.003); SRs (*M* = 0.317, s.d. = 0.585), prolific controls (*M* = 0.342, s.d. = 0.522) and database controls (*M* = 0.381, s.d. = 0.416) adopted a similar, conservative response criterion. In each case, the criterion was significantly greater than 0, indicating a general bias towards judging faces as real (SRs: (*t*_62_ = 4.309, *p* < 0.001, *d* = 0.543); prolific controls: (*t*_60_ = 5.120, *p* < 0.001, *d* = 0.656) and database controls: (*t*_61_ = 7.210, *p* < 0.001, *d* = 0.916)).

Lastly, we analysed response times (figure 2c). There was a significant difference between the groups (*F*_2,183_ = 10.768, *p* < 0.001, *ηp*^2^ = 0.105). Prolific controls (*M* = 1649 ms, s.d. = 867 ms) responded significantly faster than both SRs (*M* = 2246 ms, s.d. = 867 ms; (*t*_122_ = 4.156, *p* < 0.001, *d* = 0.746)) and database controls (*M* = 2325 ms, s.d. = 1031 ms; (*t*_121_ = 4.208, *p* < 0.001, *d* = 0.759)), suggesting they may have been less deliberative in their responses. However, there was no significant difference between SRs and database controls (*t*_123_ = 0.469, *p* = 1.00, *d* = 0.084), indicating that this is unlikely to account for the performance differences observed between SRs and the control groups in the preceding analyses. Further supporting this interpretation, there were no significant correlations between participants’ response times and sensitivity scores within any group (SRs: (*r*_63_ = −0.038, *p* = 0.765); prolific controls: (*r*_61_ = 0.166, *p* = 0.201) and database controls: (*r*_62_ = −0.048, *p* = 0.712)).

#### 2.2.2. Forced-choice training

With training, synthetic face detection accuracy was 64% (s.d. = 16%) for SRs and 51% (s.d. = 15%) for prolific controls.

As participants’ decisional strategies could affect their accuracy, we analysed their sensitivity (*d'*; figure 2a). With training, SRs (*M* = 0.738, s.d. = 0.570) were significantly more sensitive than prolific controls (*M* = −0.066, s.d. = 0.504; (*t*_144_ = 7.527, *p* < 0.001, *d* = 1.247)). SRs’ sensitivity was significantly above chance (*t*_75_ = 11.292, *p* < 0.001, *d* = 1.295), suggesting that the training procedure improved synthetic face detection ability. The prolific controls’ sensitivity did not significantly differ from chance (*t*_69_ = 1.092, *p* = 0.279, *d* = 0.131).

To investigate decisional strategies, we next analysed criterion (*C;* figure 2b). SRs (*M* = −0.023, s.d. = 0.328) and prolific controls (*M* = −0.004, s.d. = 0.303) adopted a similar criterion (*t*_144_ = 0.372, *p* = 0.710, *d* = 0.062). With training, both groups adopted a criterion that did not significantly differ from zero (SRs: (*t*_75_ = 0.617, *p* = 0.539, *d* = 0.071); prolific controls: (*t*_69_ = 0.102, *p* = 0.919, *d* = 0.012)), suggesting the absence of a systematic bias in responding.

Lastly, we analysed response times (figure 2c). Prolific controls (*M* = 2971 ms, s.d. = 1291 ms) responded significantly faster than SRs (*M* = 4342 ms, s.d. = 1293 ms; (*t*_144_ = 6.432, *p* < 0.001, *d* = 1.066)). A significant correlation between response times and sensitivity scores was found within prolific controls (*r*_70_ = 0.321, *p* = 0.007), but not within SRs (*r*_76_ = 0.110, *p* = 0.343).

#### 2.2.3. No-training versus training

To directly compare the performance measures obtained on the no-training and training versions of the task, we ran a 2 × 2 independent ANOVA with training (no training, training) and group (SR, prolific control) as between-participant variables. We could not include database controls in this analysis, as they did not complete the training version of the task.

For *d'*, there was a main effect of group (*F*_1,266_ = 73.741, *p* < 0.001, *ηp*^2^ = 0.217), whereby SRs were more sensitive than prolific controls. There was also a main effect of training (*F*_1,266_ = 84.589, *p* < 0.001, *ηp*^2^ = 0.241), whereby trained participants exhibited superior sensitivity than non-trained participants. There was no significant interaction between group and training (*F*_1,266_ = 1.335, *p* = 0.249, *ηp*^2^ = 0.005).

For *C* scores, there was no main effect of group (*F*_1,266_ = 0.167, *p* = 0.683, *ηp*^2^ = 0.001), suggesting SRs and prolific controls generally exhibited similar decisional biases. However, there was a main effect of training (*F*_1,266_ = 40.426, *p* < 0.001, *ηp*^2^ = 0.132), whereby trained participants were significantly less likely to judge that a target face was real than non-trained participants. There was no significant interaction between group and training (*F*_1,266_ = 0.002, *p* = 0.962, *ηp*^2^<0.001). Note that the effect of training on criterion does not account for the effect of training on sensitivity—these are separable constructs within signal detection theory.

For response times, there were main effects of group (*F*_1,266_ = 54.527, *p* < 0.001, *ηp*^2^ = 0.170), with SRs tending to respond more slowly than prolific controls, and of training (*F*_1,266_ = 164.660, *p* < 0.001, *ηp*^2^ = 0.382), with trained participants responding more slowly than non-trained participants. A significant interaction between group and training was observed (*F*_1,266_ = 8.432, *p* = 0.004, *ηp*^2^ = 0.031), indicating that training disproportionately slowed responses among SRs.

## 3. Experiment 2

We next investigated the discrimination of synthetic from real faces when two images were presented simultaneously (one real, one synthetic) in a 2AFC procedure.

### 3.1. Methods

#### 3.1.1. Participants

We recruited a new sample of SRs, prolific controls and database controls (table 2), using the same criteria as experiment 1. We used the same power analysis as experiment 1 and aimed to recruit at least 60 participants per condition; we succeeded in each condition aside from the database control sample (for which we recruited 54 participants). The majority of participants self-identified as White (SRs = 79.9%, prolific controls = 73.1%, database controls = 88.9%), followed by Black (SRs = 1.4%, prolific controls = 12.7%, database controls = 1.9%), Asian (SRs = 3.5%, prolific controls = 8.2%, database controls = 5.6%) and mixed (SRs = 9.7%, prolific controls = 3%, database controls = 0%). A small number of participants (SRs = 5.6%, prolific controls = 3%, database controls = 3.7%) selected ‘other’ or preferred not to disclose their ethnicity.

**Table 2.** Group demographics and performance on face perception and recognition ability screening tests for the final samples. The number of participants (*n*) on a particular test is given if they differ from the overall *N.* CFMT, Cambridge Face Memory Task Long form [24]; GFMT, Glasgow Face Memory Task [39]; KFMT, Kent Face Matching Task [40]; AFMT, Adult Face Memory Test [41]. ***p < 0.001; *p < .05; n.s: non-significant. Across tasks, the SR groups were well-matched. SRs in the no-training task were significantly better at the KFMT and AFMT than those in the training task (*p*s < 0.01); all other effects were non-significant. Our prolific controls were also well-matched, although those in the no-training task were significantly younger than those in the training task (*p* = 0.02); all other effects were non-significant.

		*N*	gender	age (s.d.)	CFMT+ (s.d.)	GFMT (s.d.)	KFMT (s.d.)	AFMT (s.d.)
2AFC task	super-recognizers (SR)	69	F: 48 M: 21	41.28 (8.31)	96.99 (2.17)	39.72 (0.62)	35.06 (2.03)	55.58 (2.42)
	prolific controls (PC)	65	F: 34 M: 30 other: 1	37.92 (11.45)	—	—	—	—
	database controls (DC)	54	F: 31 M: 20 other: 3	45.11 (10.25)	73.30 (10.01)	33.87 (2.04)	26.48 (2.79)	43.98 (4.07)
	between group difference		*n.s*.	DC > PC ***	SR > DC ***	SR > DC ***	SR > DC ***	SR > DC ***
2AFC training	super-recognizers	75	F: 51 M: 24	42.27 (8.19)	96.96 (2.40)	39.53 (.78)	*n* = 74 34.01 (2.38)	53.15 (4.44)
	prolific controls	69	F: 34 M: 34 other: 1	42.55 (10.21)	—	—	25.62 (3.90)	—
	between group difference		SR > PC *	*n.s*.	—	—	SR > PC ***	—

#### 3.1.2. Design and procedure

Experiment 2 consisted of a 2AFC experiment, where two stimuli were randomly assigned to each side of the screen per trial (one synthetic, one real), and participants were required to indicate which face was ‘not real’. The synthetic and real face images presented on each trial were selected such that they appeared to be the same gender and ethnicity. This 2AFC task is best considered as a discrimination task, rather than a detection task (e.g. experiment 1), as observers were required to discriminate between the two stimuli presented simultaneously. Performance on this task is unlikely to be driven by decisional bias, as the signal is always present and observers are always indicating that one of the images is ‘not real’. The training version of the task followed the same training procedure described above.

#### 3.1.3. Data analysis

Participants were included if they completed 3 out of 5 catch trials accurately. In the non-training task, four SRs, six database controls and 10 prolific controls were excluded on this basis. In the training task, seven SRs and 15 prolific controls were excluded on this basis. The participants described in table 2 are the final samples, after these exclusions had taken place. For ease of comparison with experiment 1, we also calculated sensitivity for the 2AFC task. The signal detection measures *d*′ and *C* cannot be calculated in the traditional way (as in experiment 1) for these data as each response is a single response to two images; therefore, we cannot calculate hits and false alarms for synthetic faces or real faces. Perceptual bias in this task is driven by observers preferring to select the image on one side of the screen, so it is not related to discrimination criteria. Therefore, we calculated unbiased *d*' in MATLAB using the Palamedes toolbox [47], this time directly from the percent correct accuracy scores.

### 3.2. Results

#### 3.2.1. Two-alternative forced choice task

Accuracy on the task was low for all three groups: SRs = 54% (s.d. = 20%), prolific controls = 42% (s.d. = 15%) and database controls = 42% (s.d. = 11%), although somewhat higher than experiment 1.

**Figure 3.**
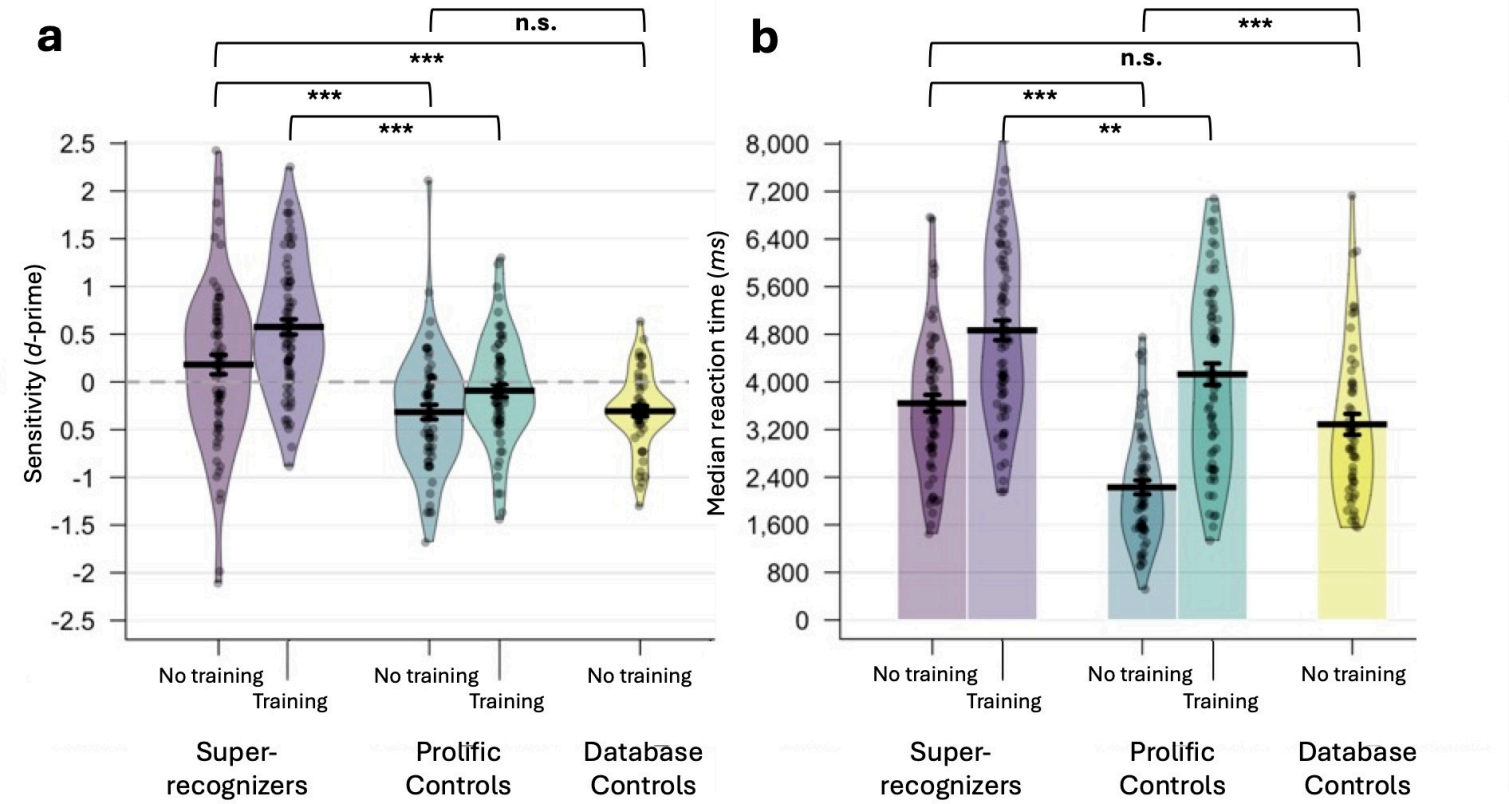
Sensitivity discriminating synthetic faces (a) and response times (b) for the 2AFC task and the 2AFC training task across the five participant groups. Note that the no-training and training tasks were run on independent sets of participants. ***p < 0.001; **p < 0.01; n.s.: non-significant. Error bars: standard error of the mean; dashed line: chance performance.

We analysed participants’ sensitivity (*d*'; figure 3a) and found a significant difference in *d*' scores between the groups (*F*_2,185_ = 12.058, *p* < 0.001, *ηp*^2^ = 0.115). SRs (*M* = 0.182, s.d. = 0.849) were significantly more sensitive than both prolific controls (*M* = −0.317, s.d. = 0.614; (*t*_132_ = 3.873, *p* < 0.001, *d* = 0.669)) and database controls (*M* = −0.307, s.d. = 0.404; (*t*_121_ = 3.898, *p* < 0.001, *d* = 0.708)). No significant difference was found between the control groups (*t*_117_ = 0.097, *p* = 1.00, *d* = 0.018).

SRs’ sensitivity did not differ significantly from chance (*t*_68_ = 1.776, *p* = 0.080, *d* = 0.214), indicating that they were not reliably distinguishing synthetic from real faces. By contrast, the sensitivity scores of both control groups fell significantly below chance—again, consistent with susceptibility to AI hyperrealism (prolific controls: (*t*_64_ = 4.158, *p* < 0.001, *d* = 0.516); database controls: (*t*_53_ = 5.593, *p* < 0.001, *d* = 0.761)).

Next, we analysed response times (figure 3b). There was a significant difference between the groups (*F*_2,185_ = 27.441, *p* < 0.001, *ηp*^2^ = 0.229). Prolific controls (*M* = 2229 ms, s.d. = 944 ms) responded significantly faster than both SRs (*M* = 3642 ms, s.d. = 1174 ms; (*t*_132_ = 7.648, *p* < 0.001, *d* = 1.322)) and database controls (*M* = 3287 ms, s.d. = 1293 ms; (*t*_117_ = 5.150, *p* < 0.001, *d* = 0.948)), suggesting they may have been less deliberative in their responses. However, there was no significant difference between SRs and database controls (*t*_121_ = 1.590, *p* = 0.228, *d* = 0.289), indicating that this is unlikely to account for the performance differences observed between SRs and controls in the preceding analyses. There were no significant correlations between participants’ response times and sensitivity scores within the SR (*r*_69_ = −0.133, *p* = 0.277) and prolific control groups (*r*_65_ = −0.124, *p* = 0.323); however, this correlation was significant within database controls (*r*_54_ = 0.306, *p* = 0.025)).

#### 3.2.2. Two-alternative forced choice training

With training, accuracy was 64% (s.d. = 17%) for SRs, and 48% (s.d. = 15%) for prolific controls.

We analysed participants’ sensitivity (*d*'; figure 3a) and found that SRs (*M* = 0.576, s.d. = 0.692) were significantly more sensitive than prolific controls (*M* = −0.092, s.d. = 0.560; (*t*_142_ = 6.333, *p* < 0.001, *d* = 1.056)). SRs’ sensitivity was significantly above chance (*t*_74_ = 7.214, *p* < 0.001, *d* = 0.833), whereas prolific controls’ sensitivity did not significantly differ from chance (*t*_68_ = 1.361, *p* = 0.178, *d* = 0.164).

We then analysed response times (figure 3b). Prolific controls (*M* = 4129 ms, s.d. = 1509 ms) responded significantly faster than SRs (*M* = 4867 ms, s.d. = 1432 ms; (*t*_142_ = 3.009, *p* = 0.003, *d* = 0.502)). No significant correlation between response times and sensitivity scores was found within either group (SRs: (*r*_75_ = −0.216, *p* = 0.063); prolific controls (*r*_69_ = 0.050, *p* = 0.685).

#### 3.2.3. No-training versus training

To directly compare the performance measures obtained on the no-training and training versions of the task, we ran a 2 × 2 independent ANOVA with training (no training, training) and group (SR, prolific control) as between-participant variables.

For sensitivity, there was a main effect of group (*F*_1,274_= 49.715, *p* < 0.001, *ηp*^2^ = 0.154), whereby SRs exhibited superior ability to discriminate synthetic faces from real faces than prolific controls. There was also a main effect of training (*F*_1,274_ = 14.029, *p* < 0.001, *ηp*^2^ = 0.049), whereby trained participants exhibited superior sensitivity than non-trained participants. There was no significant interaction between group and training (*F*_1,274_ = 1.051, *p* = 0.306, *ηp*^2^ = 0.004).

For response times, there were main effects of group (*F*_1,274_ = 48.009, *p* < 0.001, *ηp*^2^ = 0.149), with SRs tending to respond more slowly than prolific controls, and of training (*F*_1,274_ = 101.434, *p* < 0.001, *ηp*^2^ = 0.270), with trained participants responding more slowly than non-trained participants. A significant interaction between group and training was observed (*F*_1,274_ = 4.734, *p* = 0.030, *ηp*^2^ = 0.017), indicating that training disproportionately slowed responses among prolific controls.

## 4. Additional analyses

### 4.1. Effects of ethnicity

Although not a focus of the current experiment, our stimuli included faces that were both White and non-White. Additional descriptive statistics, broken down by stimulus ethnicity, participant ethnicity (defined as White and non-White), and task, are given in the electronic supplementary material, S2. To summarize these descriptive statistics, the pattern of effects reported in the preceding analyses is largely consistent. However, there was a general tendency for participants—regardless of their own reported ethnicity—to exhibit greater sensitivity at detecting synthetic faces when these identities were non-White compared to White. This is in line with previous findings [1].

### 4.2. Effects of low-level stimulus properties

As previously mentioned, we observed a difference between the stimuli in their level of saturation, with real face images being more saturated than synthetic face images. To investigate whether saturation level was related to participants’ accuracy, we conducted additional analyses (electronic supplementary materials, S3). In brief, we found little evidence that the images’ saturation levels were being used by some/all participants to complete the tasks.

### 4.3. Items analysis

To explore whether the observed training effects applied to a minority of images or were evident for all synthetic images, we plotted the training effect on image accuracy (trained – untrained percentage accuracy rates) for SRs and prolific controls. Taking this approach meant that approximately 60 observations contributed to the mean accuracy of each item.

**Figure 4.**
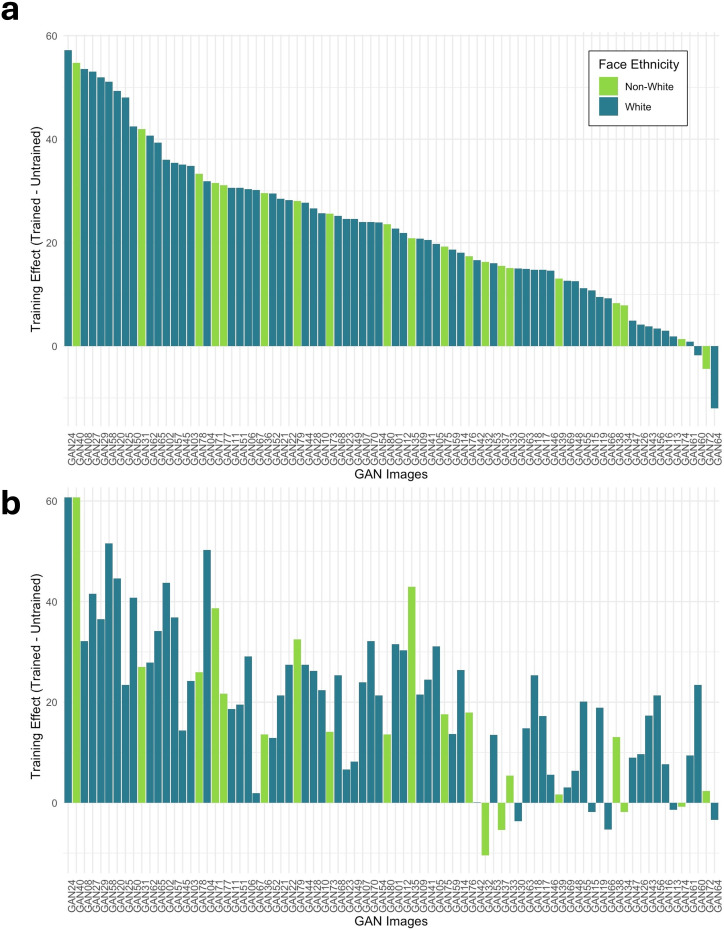
Training effect on image percentage accuracy for super-recognizers (a) and prolific controls (b) for White and non-White faces. Synthetic face images are ordered by the magnitude of the training effect in super-recognizers.

For the forced-choice task, we found that some images benefited more from training than others (figure 4). Images that benefited from training were not necessarily the same in the SR and the prolific control group. In both groups, the majority of images benefited from training by more than 10% (SRs: 55 out of 80; prolific controls: 47 out of 80), and many benefited by more than 20% (SRs: 45 out of 80; prolific controls: 41 out of 80). For a small number of images (SRs: *n* = 3; prolific controls: *n* = 9), training decreased accuracy. To explore whether training effects were most likely driven by non-White faces, we coded the effect of training depending on whether the synthetic face presented was non-White or White, with no systematic differences being clear.

**Figure 5.**
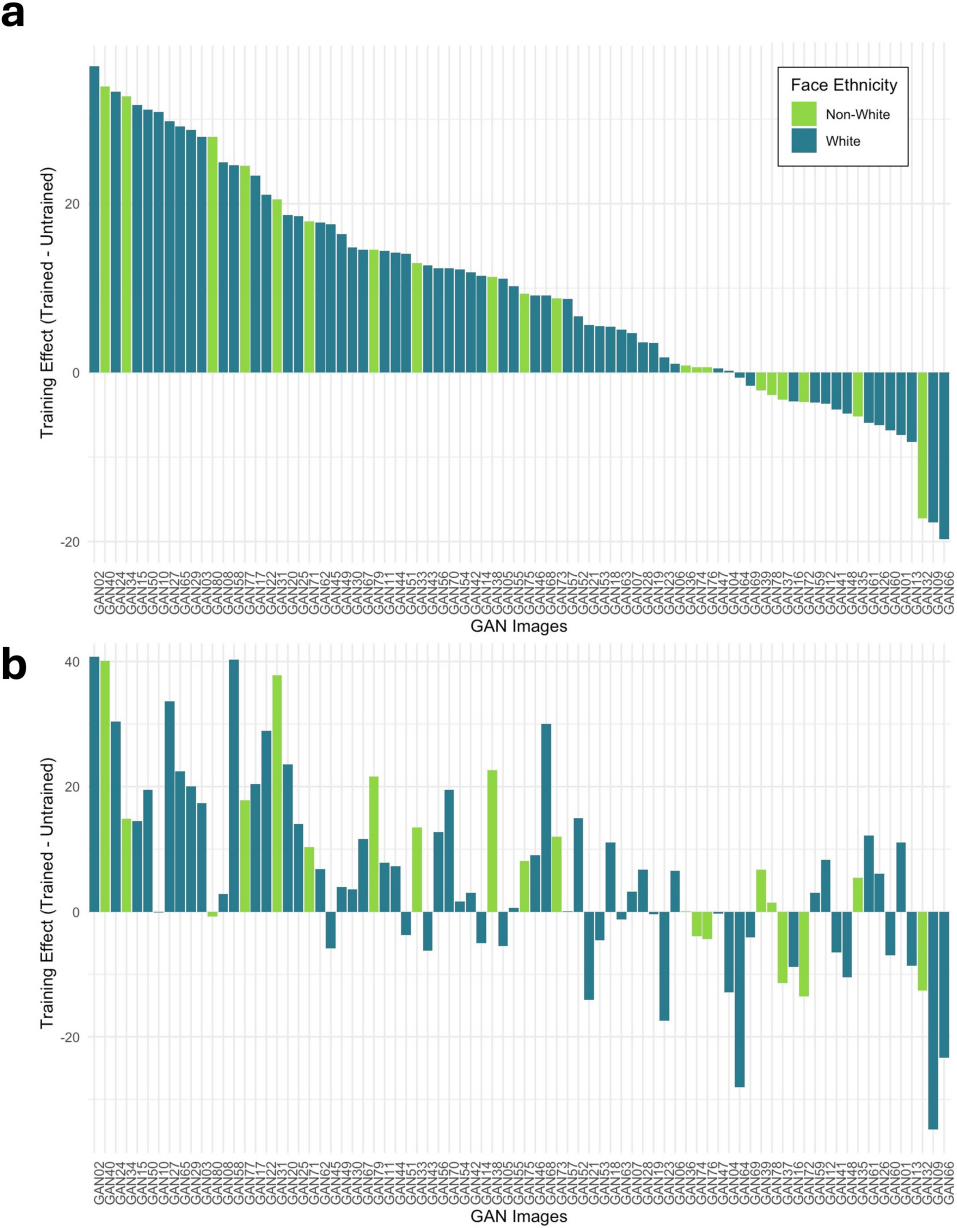
Training effect on image percentage accuracy for super-recognizers (a) and prolific controls (b) for White and non-White faces. Synthetic face images are ordered by the magnitude of the training effect in super-recognizers.

In the 2AFC task (figure 5), this measure is affected by the real face images in a way that is not the case for the forced-choice task, as each trial contains both a synthetic and a real image. Images that benefited from training were not necessarily the same in the SR and prolific control group, and some images benefited more from training than others. In the SR group, around half of the images benefited from training by more than 10% (40 out of 80), whereas only around a third of images benefited from training by more than 10% in the prolific control group (30 out of 80). Training decreased accuracy for a number of images (SRs: *n* = 20; prolific controls: *n* = 28) in the 2AFC task. Again, the training effect did not appear to systematically affect White or non-White face images.

## 5. General discussion

We investigated SRs' and typical-ability control participants’ ability to (i) detect and (ii) discriminate synthetic faces from real faces. New samples of SRs and typical ability control participants were then given a training task and completed the same detection and discrimination tasks. In the first experiment, we used a task where participants were presented with only one face on each trial and asked to detect whether it was ‘real’ or ‘not real’. In the second experiment, we used a discrimination task, where both a real face and a synthetic face were simultaneously presented on each trial and participants were asked to select which face was ‘not real’. In both tasks, SRs outperformed typical-ability control participants, although the groups performed at and below chance, respectively. We then introduced a training procedure to the start of both experiments and collected new samples of participants. In this training procedure, we pointed out potential rendering artefacts and gave trial-by-trial feedback on a block of trials. Trained SRs and controls had significantly better performance than those without training, and the magnitude of the training effect was similar in both groups.

In the non-training tasks, SRs’ performance was around chance level; however, they performed significantly better than control participants. Our control participants performed significantly below chance, showing evidence of AI hyperrealism in their responses, where synthetic faces tended to be perceived more real than real faces. Participants performed worse on our task compared to previous studies (e.g. [20]), probably owing to differences in stimuli. We used the latest iteration of StyleGAN3, whereas previous research used its predecessor StyleGAN2 (e.g. [3,14,20]). Consistent with this, the AI hyperrealism effect was also larger in our study than others (e.g. [20]), and our SRs performed at chance, whereas previous results have shown SRs’ performance to be better than chance [20]. This indicates that developments in GANs are predictably making synthetic faces look more realistic and are therefore less easy to detect. As a research field, we must attempt to keep up with the developments in synthetic face generation by using the most up-to-date GAN face stimuli in our experiments.

Trained SRs performed better than chance, whereas trained control participants’ performance was not significantly different from chance. As trained control participants’ sensitivity was not significantly below chance, our training eliminated the AI hyperrealism effect. The success of our training technique relies on the presence of artefacts in the images. The majority of previous training studies using synthetic faces have also relied on GAN artefacts (e.g. [3,14,15]). The need to detect image manipulations is not unique to fully synthetic GANs. Morphed face images—where images of different people are combined into a single image for the purpose of allowing multiple people to use the image in formal documentation—are also important to detect. Previous attempts at training observers to detect morphed face images have only succeeded when obvious image artefacts were present in the images. Robertson *et al.* [48] did not remove artefacts such as blurred hair and jawlines and found that training focused on identifying these artefacts improved detection. Conversely, Kramer *et al.* [49] removed these artefacts and focussed training on more subtle artefacts such as skin smoothness and did not find a training effect. As GAN systems improve, the artefacts remaining in images are likely to become increasingly less obvious; therefore, training effects may become smaller. Our research suggests that training SRs to identify synthetic faces could help overcome this issue.

Overall, SRs were better at the tasks than typical-ability participants, but the typical-ability participants were at chance at best. SRs outperform typical ability control participants on a range of different face tasks [28,30,32–35], and also non-face tasks [28,29], suggesting that they have better perceptual abilities across domains. SRs may process local information differently from controls [50], whilst evidence suggests that they use faces’ spatial frequency information similarly to controls, albeit more consistently [51]. It is possible that without training, SRs are better than controls at detecting GAN rendering artefacts. However, we think this is an unlikely mechanism of the group effect, as our training procedure, which was specifically targeted at identifying GAN face rendering artefacts, had a similar impact on SRs’ and controls’ performance. If SRs were already attuned to these rendering artefacts, we would have expected the training effect to be smaller in SRs than controls. An alternative possibility is that SRs use other aspects of their face perception abilities to detect synthetic faces. Interestingly, Dunn *et al*. [20] found that SRs were less likely to use familiarity and memorability to drive their judgements than control participants and were thus less likely to be misled by these cues. This suggests that SRs are naturally using different face-related cues than controls to detect synthetic faces, but both groups’ abilities can be enhanced with specific training that targets a different set of detection cues (e.g. rendering artefacts). The finding that SRs’ ability can be enhanced with training is positive, as it suggests that we can combine training with ability to improve synthetic face detection.

We found similar effects in both tasks, despite them requiring different decisions. In the training tasks, both versions were preceded by training that was in the format of a forced-choice task. As the training for both the forced-choice and the 2AFC task worked well, this shows that our training can be generalized to different task designs. We also found that the training effect tended to be evident for the majority of synthetic images, rather than just a small selection of the images with more obvious artefacts. Recently, a study has developed a training procedure that has a large effect size on synthetic face detection in typical ability groups, with the training effect lasting at least 20 days [16]. Future experiments could further elucidate and target the most effective aspects of training for SRs and test the extent to which the training generalizes to different tasks and is maintained over time.

An interesting pattern in the data was that SRs took longer to respond on the tasks than prolific controls, as in most research, SRs have tended to provide faster and more accurate responses than controls (e.g. [27]). In addition, previous research on deepfake detection found that accuracy and response times were negatively associated, such that an increase in response times was associated with a decreased likelihood of making a correct response [37]. One might assume that SRs’ superior synthetic face detection and discrimination were therefore driven by general factors such as greater conscientiousness, deliberation, or scrutinization of the target faces, rather than their superior face processing abilities. However, two aspects of our data challenge this interpretation. First, while SRs’ reaction times differed significantly from the prolific control group, they did not differ from the database control group—despite SRs outperforming both control groups on the tasks in terms of accuracy and sensitivity. Moreover, prolific controls and database controls achieved comparable levels of performance despite varying significantly in response speed. This makes it difficult to argue that slower responses alone explain SRs' performance advantage. Second, correlations between response times and sensitivity were generally not significant within each of the participant groups and for either task format. This suggests that prolonged response time was not reliably associated with better synthetic face detection/discrimination and thus cannot explain the group differences in performance observed.

Our SRs were recruited based on their scores on at least three objective face recognition tests, where they were required to be at least 2 s.d. above the mean on three of the tests, and at least 1.5 s.d. above the mean on a fourth test, if they had completed a fourth test. All had completed the CFMT+ [24], and the GFMT [39]; most had also completed the KFMT [40] and the AFMT [41]. While some previous research has used performance on one test [25,28] to screen SRs, newer recommendations suggest superior performance should be demonstrated on at least two out of three face processing tasks [52]. The CFMT+ is recommended [52,53], as is the cut-off of 2 s.d. above the mean [53]. We used the GFMT, which tends to have ceiling effects [54], and is now not recommended for SR screening [52,53]. Where we have scores on additional tests (including the more sensitive GFMT2 [54]), we have included these in our data repository. The trained SRs tended to have slightly (but significantly) lower scores on some of the face processing screening tests than the non-trained SRs. This supports that our training was effective, as the results cannot be attributed to better face recognition ability in the trained SR groups. We believe that had our screening procedure been more conservative, we may have found larger effects between SRs and typical ability control participants.

Our stimuli were composed of faces from different ethnicities. This was purposeful, as we wanted to portray a realistic face-diet. We did not set out to systematically investigate demographic effects in our experiments. However, individuals’ ability to recognize faces can be impacted by ethnicity, such that own-ethnicity faces are better recognized than faces from 'other’ ethnicities [55–57]. In addition, synthetic faces have tended to be trained primarily on White faces, such that their rendering of non-White faces may be less likely to be perceived as realistic [1]. Descriptive statistics for the different participant ethnicity groups (White, non-White) and stimulus ethnicity (White, non-White) are provided in the electronic supplementary material. There is some suggestion from these that non-White synthetic faces may have been easier to detect and discriminate than White faces, as indicated by previous research [1]. Our inclusion of non-White faces could therefore have made our tasks easier than tasks that have only used White faces (e.g. [1,20]), making the fact that our tasks were more difficult than these studies particularly striking.

In the future, our brief and easily implementable training procedure could be combined with another intervention or effect, such as wisdom of the crowds [17–19], to further improve SRs’ performance. As previously noted, our training cannot be considered a lasting intervention, as we have not yet completed a follow-up; this will be an important next step. An exciting and currently unexplored possibility is using trained SRs to interpret the output from AI detection algorithms, which could have a large impact on real-world synthetic face detection scenarios. For example, to best detect synthetic faces, it may be possible to use AI detection algorithms with a human-in-the-loop approach – where that human is a trained SR.

In conclusion, we found that detecting and discriminating synthetic faces is an extremely difficult task, for which typical-ability participants perceive synthetic faces as more real than real faces. SRs consistently outperformed typical-ability participants, where without training they were less susceptible to this AI hyperrealism effect. A training and feedback procedure was able to increase performance to a similar extent for both SRs and typical-ability participants. Our results suggest that SRs are using cues unrelated to rendering artefacts to detect synthetic faces. The performance of trained SRs could be harnessed for real-world applications, such as online identity verification.

## Data Availability

Data are available on OSF [58]. Supplementary material is available online [59].
